# Clinical method evaluation of hemoglobin S and C identification by top-down selected reaction monitoring and electron transfer dissociation

**DOI:** 10.1186/s12014-019-9261-1

**Published:** 2019-12-17

**Authors:** Olivier Lassout, Ralf Hartmer, Wolfgang Jabs, Lorella Clerici, Yury O. Tsybin, Kaveh Samii, Nicolas Vuilleumier, Denis Hochstrasser, Alexander Scherl, Pierre Lescuyer, Didia Coelho Graça

**Affiliations:** 1 Department of Medical Specialties, Faculty of Medicine, University of Geneva, Geneva, Switzerland; 2 Division of Laboratory Medicine, Department of Laboratory Medicine, Genetics and Pathology, Geneva University Hospitals, 4 Rue Gabrielle-Perret-Gentil, 1205 Geneva, Switzerland; 3 Bruker Daltonics, Bremen, Germany; 4 Spectroswiss, EPFL Innovation Park, Lausanne, Switzerland; 5 Division of Hematology, Geneva University Hospitals, Geneva, Switzerland

**Keywords:** Top-down, ETD, Electron transfer dissociation, Intact protein, Hemoglobin, Hemoglobin disorders, Hemoglobin variant, SRM, Selected reaction monitoring

## Abstract

**Background:**

Biological diagnosis of hemoglobin disorders is a complex process relying on the combination of several analytical techniques to identify Hb variants in a particular sample. Currently, hematology laboratories usually use high-performance liquid chromatography (HPLC), capillary electrophoresis and gel-based methods to characterize Hb variants. Co-elution and co-migration may represent major issues for precise identification of Hb variants, even for the most common ones such as Hb S and C.

**Methods:**

We adapted a top-down selected reaction monitoring (SRM) electron transfer dissociation (ETD) mass spectrometry (MS) method to fit with a clinical laboratory environment. An automated analytical process with semi-automated data analysis compatible with a clinical practice was developed. A comparative study between a reference HPLC method and the MS assay was performed on 152 patient samples.

**Results:**

The developed workflow allowed to identify with high specificity and selectivity the most common Hb variants (Hb S and Hb C). Concordance of the MS-based approach with HPLC was 71/71 (100%) for Hb S and 11/11 (100%) for Hb C.

**Conclusions:**

This top-down SRM ETD method can be used in a clinical environment to detect Hb S and Hb C.

## Background

Hemoglobin (Hb) is a tetrameric blood protein contained in red blood cells, which carries oxygen through all organs and tissues. The Hb protein structure is defined by four subunit proteins (chains) that form the tetramer via non-covalent binding: α, β, δ, and γ chains. In normal situation, an adult person has approximately 98% of Hb A (two α chains and two β chains), 2.5 to 3.5% of Hb A_2_ (two α chains and two δ chains) and less than 1% of Hb F (two α chains and two γ chains). A pathological situation can be observed in two major cases. First, a production of an abnormal Hb chain leading to a qualitative disorder (i.e. sickle cell disease with Hb S variant). Second, an unbalanced production between different chains (i.e. unbalanced α/β chain ratio) leading to a quantitative disorder, called thalassemia. Until now, a large number of Hb variants (more than 1300) have been described but just a few of them cause clinical manifestations [1–3].

Hemoglobin disorders diagnosis is a complex process based on the combination of clinical and biological data. Typically, the process starts with patient information (i.e. ethnic origin), clinical history and hematological data (i.e. Hb levels, red blood cell morphology) which serves as a first indicator for a hemoglobin disorder [4]. Then, the relative percentage of Hb A, Hb A_2_ and Hb F is determined by cation exchange high-performance liquid chromatography (CEX-HPLC) or capillary electrophoresis (CE) methods with UV detector for both methods [5]. The presence of an Hb variant is usually detected at this step. Unfortunately, due to the limited selectivity of CEX-HPLC and CE methods, only a presumptive Hb identification can be performed and a combination with other methods is mandatory to allow a correct Hb variant characterization [5]. Identification of the most common and clinically significant Hb variants (i.e. Hb S, Hb C, Hb E, Hb D-Punjab and Hb O-Arab) faces the same problem. They are all the result of a single point mutation on the Hb β chain. Hb S and Hb C result from the substitution of valine or lysine, respectively, instead of glutamic acid at position 6 on β chain; Hb E from the substitution of lysine instead of glutamic acid at position 26 on β chain, Hb D-Punjab and Hb O-Arab from the substitution of glutamine or lysine, respectively, instead of glutamic acid at position 121 on β chain.

Today, in hematology laboratories, techniques commonly used to separate Hbs and identify Hb variants are based on charge and/or size differences (e.g. CEX-HPLC and/or different electrophoretic techniques). In this context, mass spectrometry (MS) could represent an optimal tool for Hb disorders diagnosis by measuring Hb mass to charge ratio (i.e. *m/z*), known to be a highly specific and sensitive molecular signature. Promising preliminary data indicate that several Hb variants can be detected and identified on the basis of their *m/z* difference, and that this orthogonal information has the potential to provide additional information over classical electrophoretic methods and to improve the turn-around time (TAT). Several MS methods have already been proposed in this context using different bottom-up and top-down (TD) approaches with electrospray ionization (ESI) or matrix-assisted laser desorption ionization (MALDI) approaches, as well as low or high-resolution mass spectrometers [6–10]. Among these, the top-down (TD) MS approach using electron transfer dissociation (ETD) as the method of precursor ion fragmentation in the gas phase has many advantages. Firstly, it allows circumventing the variability resulting from protein digestion and is therefore more adapted to routine laboratory environment by decreasing analytical variability and facilitating sample handling [11]. Secondly, when coupled to selected reaction monitoring (SRM), TD ETD method has shown to be able to detect selectively Hb S and Hb C [12].

However, such a TD ETD MS approach is not yet available in routine hematological laboratories. Therefore in this works, we developed a targeted high-throughput TD ETD MS approach coupled to data analysis allowing result interpretation by users with no specific expertise in MS. We evaluated the concordance of the TD ETD MS method with standard CEX-HPLC diagnostic procedures to identify Hb A, Hb C, Hb E variants, as the most frequent Hb disorders encountered in routine hematology diagnostics.

## Methods

### Reagents

The reagents used in this study were as follows: acetonitrile (HPLC–MS grade), isopropanol (HPLC–MS grade) and water (HPLC–MS grade) were from Romil Ltd (Cambridge, United Kingdom); formic acid (FA) (HPLC–MS grade) was from Biosolve-chemicals (Dieuze, France).

### Samples

A total of 152 consecutive EDTA whole blood samples were collected during 5 months at the Geneva University Hospitals (HUG) after completion of routine analyses by the HUG hemoglobin disorders laboratory. The sample collection was composed of 41 samples without Hemoglobinopathies, 32 with Thalassemia, 1 with Hb constant spring, 2 with Hb Lepore Boston Washington, 16 with Hb S heterozygote, 12 with Hb S homozygote without transfusion, 33 with Hb S homozygote with transfusion, 1 with Hb C heterozygote and alpha thalassemia heterozygote, 10 with Hb S and Hb C with transfusion and 4 Hb E heterozygote (Table 1). The study was approved by the ethical committee for research of the Canton of Geneva (CCER), Switzerland. As samples were processed anonymously without collection of clinical information in the context of an analytical method development, informed patient consent was not requested by the CCER.

**Table 1:**
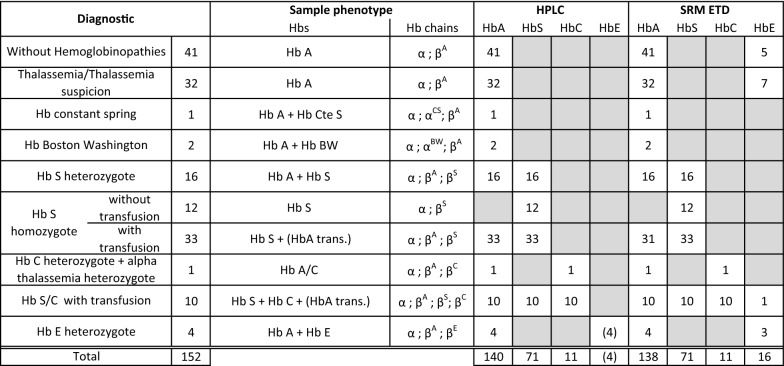
CEX-HPLC and TD SRM ETD method comparison results

Lyphochek Hemoglobin A2 Control, levels 1 and 2 (BioRad) were used as internal quality controls (IQCs). The relative percentage were 2.8 and 5.30% for HbA_2_, 2.50 and 9.70% Hb F and 0 and 28.4% for Hb S for level 1 and level 2 respectively.

### Sample preparation for MS assay

Samples were stored at 4 °C until processing. Briefly, 250 μL of EDTA blood were introduced in a 2 mL Eppendorf tube and centrifuged at 3500*g* for 10 min. The plasma was removed and 750 μL of cold 0.9% NaCl solution were added. After centrifugation at 700*g* for 10 min, the supernatant was removed and this cleaning step was repeated two more times. Finally, red blood cells were lysed by adding 1750 μL of cold deionized water. After centrifugation at 12,000*g* for 10 min, the obtained Hb solution was stored at − 80 °C. For MS analysis, 12 µL of Hb solution was diluted with 988 µL of 50% acetonitrile (ACN) and 0.1% formic acid (FA).

IQC samples were prepared as mentioned by manufacturer’s instructions and diluted with the same procedure as blood sample.

### Sample introduction system

An automated sample introduction system compatible with a clinical laboratory practice was mandatory. In our previous published work, we described a SRM ETD MS method using a nano HPLC system for sample introduction [12]. The HPLC was replaced by a LC auto-sampler unit able to work at higher flow rate (Alias, Spark Holland, Emmen, Netherlands), which was directly coupled to the ESI ion source (Bruker Daltonics, Billerica, MA). The injection volume and the flow rate were set to 200 μL and 13 μL min, respectively. The eluent solution was composed of 50% ACN and 0.1% of FA. A stable spray of 4 min was generated. After 8 min of data acquisition, a system wash was performed in four steps: (i) 1000 μL of 50% of isopropanol, followed by (ii) 1000 μL of ACN, (iii) 1000 μL of 50% of isopropanol and, finally, (iv) 1000 μL of 50% ACN and 0.1% of FA. With this optimized washing procedure, we did not identify carry over from the previous infusion experiment. Each run lasted 15 min including data acquisition and washing steps.

### Mass spectrometry

The previously published SRM ETD method [12] was adapted to the new injection approach (i.e. automated direct injection). The trap ion charge control (ICC) was set to 200,000 charges for full scan and 80,000 charges for MS/MS event. Maximum ion accumulation time, scan *m/z* range, and scan average were set for full MS scans and MS/MS scans at 50 ms and 5 ms, between *m/z* 450 to 1200 and between *m/z* 400 to 1500, and at 10 and 5, respectively. Enhanced resolution (8100 amu/s^1^) and SmartMRM mode were used. A full scan mass spectrum was acquired every 20 s.

In order to enhance the efficiency of precursor ion isolation for the SRM ETD, the optimized targeted isolation consists of two consecutive isolation steps, as previously described [13]. The optimization of the individual Hb precursor isolation results in higher actual ICC number (i.e. number of isolated precursor ions) and therefore yields in higher signal to noise ratios for the products ions selected for the present SRM-ETD transitions.

Table 2 shows the result of the optimized isolation center position and the corresponding isolation width for the first and second isolation step for each chain (Table 2). Hb A, Hb C, Hb E, Hb D-Punjab and Hb O-Arab β chain have very close molecular weights (MW) resulting in a less than 1 Da of mass shift [12]. As mass spectrometry instruments measures *m/z* ratios, the expected *m/z* difference for 19^+^ precursor ions is less than *m/z* 0.05. Consequently, a co-isolation approach was selected and the isolation parameters for Hb C, Hb E, Hb D-Punjab and Hb O-Arab were the same as Hb A (i.e. β^A^ chain isolation parameters, Table 1). ETD parameters were set at 100,000, 160 *m/z* and 60 ms for ETD ICC, low *m/z* cut-off and ion–ion reaction time, respectively. The selectivity and specificity of this identification method was provided by targeting three specific product ions for each chain except for Hb E (i.e. one specific product ion) (Table 3).

**Table 2:** Isolation parameters for α chain, Hb A and Hb S β chains. Hb C, Hb E, Hb D-Punjab and Hb O-Arab isolation is based on the same parameters as Hb A β chain

Chain	Charge state	First isolation step		Second isolation step	
		Precursor ion (*m/z*)	Isolation width (*m/z*)	Precursor ion (*m/z*)	Isolation width (*m/z*)
β^S^	[M+19H]^19+^	837	11.5	835.3	2.5
β	[M+19H]^19+^	837	11.5	837	3
α	[M+18H]^18+^	841.9	12.5	841.9	2.5

Hb C, Hb E, Hb D-Punjab and Hb O-Arab isolation is based on the same parameters as Hb A β chain

**Table 3:** SRM-ETD transition list for specific α chain and Hb A, Hb C, Hb S and Hb E β chains identification

Chain	Precursor ion (*m/z*)	Product ions					
		ion	(*m/z*)	ion	(*m/z*)	ion	(*m/z*)
α	841.9	*c*_*6*_^*1+*^	600.3	*c*_*7*_^*1+*^	728.4	*c*_*8*_^*1+*^	829.5
β	837	*c*_*6*_^*1+*^	694.4	*c*_*7*_^*1+*^	823.4	*c*_*8*_^*1+*^	951.5
β^C^	837	*c*_*6*_^*1+*^	693.4	*c*_*7*_^*1+*^	822.5	*c*_*8*_^*1+*^	950.6
β^S^	835.3	*c*_*6*_^*1+*^	664.4	*c*_*7*_^*1+*^	793.5	*c*_*8*_^*1+*^	921.5
β^E^	837	*c*_*26*_^*3+*^	921.6				

### Data analysis

**Fig. 1:**
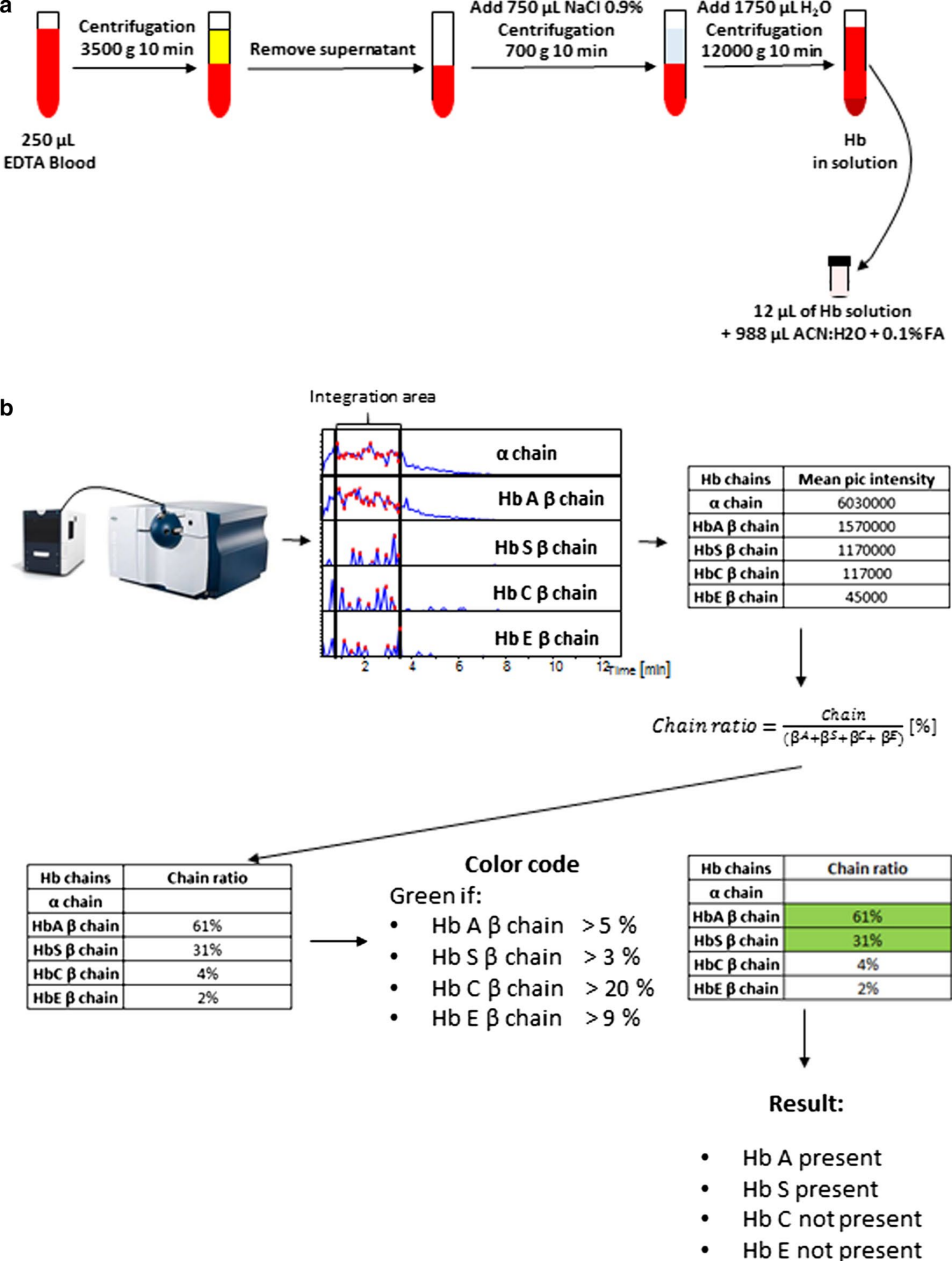
Complete workflow of the SRM ETD method. **a** Sample preparation protocol. **b** Scheme of the data acquisition by TD SRM ETD method and data analysis. Specific EIC for α chain, β^A^ chain, β^S^ chain, β^C^ chain, β^E^ chain. For each chain, the mean pic intensity is obtained and chain ratio is calculated. The detection of each chain is based on a color code and a cut-off value

We developed a script for automated generation of the extracted ion chromatogram (EIC) followed by the integration of the area under the chromatographic curve. The new automated sample introduction resulted in a stable spray during four min. The EIC corresponding to the specific transitions were generated for each chain. Peak intensities between 1 and 4 min of run time were summed and an averaged peak intensity value was obtained. The ratio of the averaged chain intensity to the sum of all non-α chains intensities were calculated and expressed in percentage (e.g. ${\text{Hb}}\;{\text{S}}\;\upbeta\;{\text{chain}}\;{\text{ratio}}\; = \;\frac{{\upbeta^{S} }}{{(\upbeta +\upbeta^{S} + \beta^{C} +\upbeta^{E} }}\;[\% ]$). Threshold values were introduced for the detection of each chain: Hb A: ratio above 5%; Hb S: ratio above 3%; Hb C: ratio above 20%; Hb E: ratio above 9%. The result was then reported as Hb present/Hb not present (Fig. 1).

### Method evaluation and comparison study design

Repeatability and reproducibility evaluation were performed by measuring four patients with different established phenotypes 20 times and during 20 days (n = 20) respectively. Sample phenotypes were determined by BioRad Variant II used as the reference method and are as follows: A/A (healthy sample, α chain and Hb A β chain); S/S (homozygote Hb S sample, α chain and Hb S β chain); C/C (homozygote Hb C sample, α chain and Hb C β chain) for reproducibility and A/C (heterozygote Hb C sample, α chain, Hb A β chain and Hb C β chain) for repeatability and A/E (heterozygote Hb E sample, α chain, Hb A β chain and Hb E β chain).

We compared results obtained by the hematology laboratory by CEX-HPLC and TD ETD MS on the same blood samples. CEX-HPLC analysis was performed on a Variant II HPLC system with Dual Kit reagents (Bio-Rad, Hercules, CA, USA) according to the manufacturer’s instructions. Results from CEX-HPLC and SRM ETD methods were independently managed and compared at the end of the study. Study design was as follows: 152 samples were analyzed in batch of 19 samples per day. Each batch was validated according with the results of the IQC materials.

For assay comparisons, we applied two-tailed Fisher’s exact test using XLSTAT software, Excel 2016 (Microsoft).

Important to note, MS sample preparation and sample analysis were performed by a laboratory technician that was not involved in the method development. This study was designed to evaluate the feasibility of performing the developed workflow in a clinical laboratory environment.

## Results

Reproducibility and repeatability study was performed with four samples (sample phenotype: Hb A/A, Hb S/S, Hb C/C (reproducibility), Hb A/C (repeatability) and Hb A/E) and results are presented in Table 4. The four different phenotypes were correctly characterized by TD SRM ETD method 20 times during 20 days. Intermediate imprecision for Hb A β chain, Hb S β chain, Hb C β chain and Hb E β chain were ≤ 10%, ≤ 3%, ≤ 8% and ≤ 19% of CV, respectively. Four different patient phenotypes samples were consecutive measurements 20 times in the same batch. Within-series variability for Hb A β chain, Hb S β chain, Hb C β chain and Hb E β chain were ≤ 5%, ≤ 3%, ≤ 7% and ≤ 28% of CV, respectively.

**Table 4:** Repeatability and reproducibility study results

	Reproducibility					Repeatability					
Sample phenotype	A/A	S/S	C/C	A/E		A/A	S/S	A/C		A/E	
Chains	β^A^ (%)	β^S^ (%)	β^C^ (%)	β^A^ (%)	β^E^ (%)	β^A^ (%)	β^S^ (%)	β^A^ (%)	β^C^ (%)	β^A^ (%)	β^E^ (%)
1	*79*	*83*	*67*	*61*	*30*	*82*	*90*	*49*	*46*	*78*	*12*
2	*82*	*84*	*62*	*63*	*27*	*88*	*90*	*48*	*46*	*72*	*10*
3	*78*	*81*	*71*	*64*	*29*	*77*	*91*	*50*	*45*	*77*	*14*
4	*75*	*78*	*64*	*63*	*28*	*85*	*94*	*47*	*50*	*74*	*14*
5	*92*	*83*	*66*	*63*	*31*	*79*	*92*	*47*	*46*	*75*	*13*
6	*66*	*82*	*55*	*68*	*28*	*88*	*99*	*48*	*49*	*78*	7
7	*75*	*81*	*65*	*67*	*28*	*82*	*98*	*45*	*51*	*86*	7
8	*82*	*85*	*68*	*57*	*26*	*89*	*95*	*45*	*53*	*84*	7
9	*77*	*86*	*58*	*58*	*30*	*84*	*95*	*47*	*48*	*87*	7
10	*93*	*80*	*65*	*60*	*32*	*85*	*98*	*42*	*53*	*83*	*11*
11	*81*	*82*	*73*	*75*	*19*	*86*	*96*	*50*	*45*	*80*	*15*
12	*69*	*77*	*66*	*70*	*22*	*81*	*91*	*47*	*48*	*84*	8
13	*79*	*85*	*62*	*56*	*37*	*82*	*96*	*50*	*41*	*84*	8
14	*77*	*86*	*65*	*66*	*20*	*75*	*91*	*52*	*42*	*78*	*14*
15	*78*	*85*	*55*	*59*	*22*	*82*	*96*	*48*	*46*	*82*	7
16	*74*	*88*	*65*	*61*	*35*	*84*	*93*	*48*	*49*	*69*	*14*
17	*86*	*82*	*57*	*56*	*29*	*89*	*100*	*44*	*54*	*81*	*11*
18	*99*	*86*	*59*	*70*	*22*	*85*	*96*	*39*	*53*	*77*	*11*
19	*85*	*85*	*58*	*67*	*21*	*92*	*99*	*55*	*40*	*77*	9
20	*90*	*82*	*57*	*63*	*23*	*80*	*98*	*46*	*50*	*80*	*12*
***Average***	81	83	63	63	27	84	95	47	48	79	10
***SD***	8	3	5	5	5	4	3	4	4	5	3
***CV***	10	3	8	8	19	5	3	7	9	6	28

Italic values indicate positive results

The comparative study was then performed on 152 samples to evaluate the TD SRM ETD MS method for Hb A, Hb S, Hb C and Hb E identification. Results of the comparison between the CEX-HPLC and MS methods are summarized in Table 1.

For Hb A, concordance of the MS-based approach with HPLC was 138/140 (98.6%) and a p-value of 0.838 (significance level < 0.05) indicated no statistically significant difference between the two methods. Hb A was properly identified in 138 samples belonging to the following categories: 41 samples with no hemoglobin disorders, 32 samples with thalassemia, 1 sample with Hb Constant Spring (CS), 2 samples with Hb Lepore Boston Washington (BW), 16 samples with heterozygote Hb S, 31 Hb S homozygote transfused, 1 Hb A/C, 10 Hb S/C with transfusion, and 4 Hb E heterozygote. The two discordant samples (Hb A detected by HPLC but not by TD SRM ETD) consisted in two Hb S homozygote transfused samples (Table 1). To note, it was decided to notify HPLC results in Table 1 following our clinical laboratory recommendations: if the patient is registered in blood transfusion program, HPLC Hb A result was notified in Table 1. If the patient was not registered in a blood transfusion program and has high values of Hb S (> 80%), HPLC Hb A result was not notified in Table 1 (probably glycated Hb S contamination).

For Hb S variants, the concordance of MS-based approach with HPLC was 71/71 (100%) and a p-value of 1.0 (significance level < 0.05) indicated no statistically significant difference between the two methods. Hb S was correctly identified in 71 samples belonging to the following categories: 16 samples with Hb A/S, 12 samples with Hb S homozygote, 33 with Hb S homozygote transfused and 10 samples with Hb S/C transfused.

For Hb C variants, the concordance of MS-based approach with HPLC was 11/11 (100%) and a p-value of 1.0 (significance level < 0.05) indicated no statistically significant difference between the two methods. Hb C was properly identified in 11 samples belonging to the following categories: 1 Hb A/C and 10 Hb S/C with transfusion.

For Hb E variant, the concordance of MS-based approach with HPLC was 16/4 and a p-value of 0.009 (significance level < 0.05) indicated statistically significant difference between the two methods. Hb E was correctly identified in 3 Hb A/E samples. TD SRM ETD method detected 16 Hb E samples, 12 were false positives results and one false negative. It is interesting to note that for the four Hb E containing samples, the result provided by the HPLC method was in fact the presence of a high level of HbA_2_ since, Hb E co-elute with Hb A_2_. In this context, the presence of Hb E by CEX-HPLC is only presumptive. Hb E suspicion must be confirmed using alternative methods such as isoelectric focusing and electrophoresis at acid pH.

Importantly, five cord bloods samples (2 Hb A/S and 3 Hb A) were analyzed and the same phenotype was detected by both methods (these results are included in Table 1 in Hb S heterozygote results and without Hemoglobinopathies results respectively).

## Discussion

**Fig. 2:**
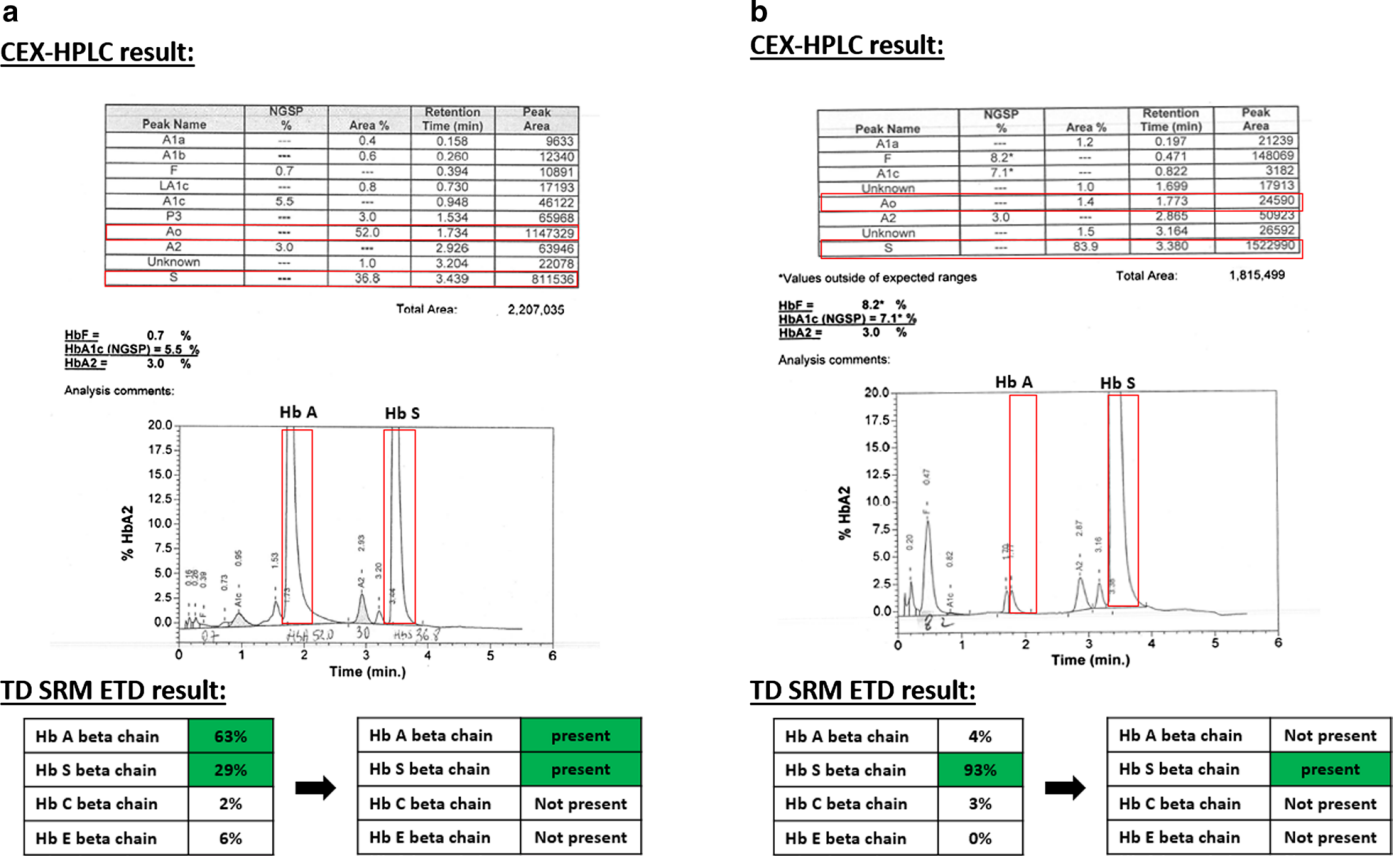
Results of CEX-HPLC method and corresponding TD SRM ETD method for two samples are presented. **a** Results of heterozygote Hb S sample. On top, results table and chromatogram are presented. Hb A is detected at 52%, 1.734 min of RT and Hb S is detected at 36.8%, 3.439 min of RT. The corresponding peaks in the chromatogram are highlighted by a red box. On bottom, TD SRM ETD results are presented in a table for Hb A, Hb S, Hb C and Hb E chains ratio and color code results. If the targeted Hb is present, the corresponding box is green. Hb A and S are detected with a ratio of 63% and 29% respectively (green boxes). **b** Results of homozygote Hb S sample. On top, results table and chromatogram are presented. Hb A is detected at 1.4%, 1.773 min of RT and Hb S is detected at 83.9%, 3.380 min of RT. The corresponding peaks in the chromatogram are highlighted by a red box. On bottom, TD SRM ETD results are presented in a table for Hb A, Hb S, Hb C and Hb E chains ratio and color code result. If the targeted Hb is present, the corresponding box is green. Only Hb S is detected with a ratio of 93% (green box)

In this comparative study to evaluate our developed MS method, a CEX-HPLC method (BioRad Variant II) was compared to a TD SRM ETD method to identify Hb A, Hb S, Hb C and Hb E in the context of hemoglobinopathies diagnosis. These two methods showed a high degree of agreement for Hb A, Hb S and Hb C identification. Important to note, HPLC method allows to obtain an overview of Hbs that are present in a sample. The developed MS method allows to detect and identify with high specificity targeted Hbs (Hb A, Hb S and Hb C). Hb A was detected by both methods in all samples except two Hb S homozygote with transfusion. For this two samples, the HPLC method detected Hb A and Hb S while the MS method detected Hb S only. This discrepancy could be explained by the fact that glycated Hb S has the same retention time (RT) as Hb A with the BioRad Variant II assay [5, 14]. Thus, a small peak eluting with the same RT as Hb A is always present for Hb S homozygote samples even if the patient was not transfused (Fig. 2b top). As the MS method is detecting Hb A and Hb S with a high specificity by combining three specific transitions, such interference is not observed with the TD ETD SRM assay (Fig. 2b bottom). We can therefore hypothesize that there was no residual Hb A from the blood donor in these two Hb S homozygote samples, explaining why the TD ETD SRM method did not detect Hb A. If confirmed, this could be a good point for the MS method as it reflects a better sample phenotype characterization compared to CEX-HPLC method in this context.

Hb S is detected by both methods in all samples carrying Hb S. Importantly; the presence of Hb S is detected and identified by TD SRM ETD method also in cord blood samples. For these samples the major Hb component is Hb F (often > 80%). Thus, Hb S and Hb A were present at low relative percentage in the analyzed samples.

Hb C was also detected by both methods for all samples carrying Hb C. One point to note is that TD SRM ETD method allows a higher selectivity and specificity for the identification of this Hb variant compared to HPLC and other protein analysis methods because the detection and identification of Hb C relies on the detection of three specific product ions.

As explained above, Hb E detection by CEX-HPLC assay is detected as being Hb A_2_. In fact, this hemoglobin has the same RT as Hb A_2_ and the presence suspicion confirmation of Hb E relies on the combination of HPLC data and other methods. For this hemoglobin variant, several false positive and one false negative results were obtained by TD SRM ETD method and repeatability result was not satisfactory (Tables 1, 4). This performance is not acceptable for clinical laboratory practice. Two factors can explain this unsatisfactory result. First, the detection of Hb E relies only on one single transition, which is not sufficient for confident detection and identification. Several investigations were made to find more specific transitions for this Hb without success. In fact, due to this mutation position on β chain (position 26), the mass shift (less than 1 Da) and the instrument resolution, there was only three theoretical transitions for this Hb. Second, this low performance could be also related to the fact that samples were analyzed by MS at least 1 year and half after collection. Some Hb degradation leading to reduced Hb levels could have therefore impacted Hb E detection. The effect of degradation could be more important for Hb E compared to other Hbs as identification of this Hb variant relied on one single transition. Therefore, other strategies should be tested to enhance the selectivity and specificity of the method for this Hb variant.

Samples carrying other Hb variants were also tested such as Hb Lepore Boston Washington and Hb Constant Spring. TD SRM ETD method successfully detected the presence of Hb A in these samples. However, these variant were not detected as this method do not allow to provide an overview of Hbs that are present in a sample (i.e. targeted method).

Concerning MS method feasibility evaluation on clinical laboratory environment, the workflow was easily executed by a laboratory technician that was not involved in the method development. The sample preparation protocol is similar to protocol used in hematological laboratories for gels methods: RBC washing procedure and lysed by water. Hb solution was then directly diluted into HPLC vials. Maximum 20 min are needed to run and perform data analysis per sample. The data analysis and interpretation is very easy to perform because it relies on the interpretation of a color code (i.e. green light if the targeted Hb is present). These results suggested that this method can be compatible with a clinical laboratory practice to identify with high specificity Hb S and Hb C.

## Conclusion

A TD MS method based on SRM ETD was compared to a commercial CEX-HPLC method (BioRad Variant II) by analyzing 152 patient samples. Results showed a satisfactory concordance between TD SRM ETD method and CEX-HPLC method for Hb A, Hb S and Hb C variants. Two transfused Hb S homozygote samples were detected with Hb A by CEX-HPLC method and without Hb A by TD SRM ETD. This discordance can be theoretically explained by the fact that glycated Hb S has the same RT as Hb A. A more specific study should be done to evaluate the performance of TD SRM ETD method compared to HPLC method to characterize transfused Hb S samples. Hb C is detected with high specificity by TD SRM ETD method which is different of CEX-HPLC. For this Hb variant, hematology laboratory had to perform several methods in addition to HPLC to confirm the presence of Hb C. On the other hand, selectivity of Hb E identification by TD SRM ETD is not satisfactory as several false positives and one false negative results were obtained. Improvements of Hb E identification should be implemented. Four cord blood samples were analyzed and correctly characterized by TD SRM ETD. A study on the analysis of newborn samples by the TD SRM ETD method will be performed and dried blood spots (DBS) will be evaluated as sample collection approach. Furthermore, validation on fresh blood samples and simplification of the sample preparation protocol will be performed in the future. This will presumably reduce signal variability. The threshold values for Hbs detection should be optimized. Finally, data analysis is currently semi-automated. Results from the developed script are manually transferred into an excel file to complete data analysis. Informatics development should be added to allow a fully automated data analysis. A completely automated data analysis and the use of DBS should allow to use the developed method for the neonatal screening of Hb S.

## Data Availability

http://hyperion.unige.ch/download/Didia_27102018.zip

## Abbreviations

BW: Lepore Boston Washington
CCER: ethical committee for research
CE: capillary electrophoresis
CEX-HPLC: cation exchange high performance liquid chromatography
CS: Constant Spring
DBS: dried blood spot
EIC: extracted ion chromatogram
ETD: electron transfer dissociation
Hb: hemoglobin
HPLC: high performance liquid chromatography
HUG: Geneva University Hospital
ICC: ion charge control
QC: quality control
MS: mass spectrometry
RT: retention time
SRM: selected reaction monitoring
